# Thyme and Savory Essential Oil Efficacy and Induction of Resistance against *Botrytis cinerea* through Priming of Defense Responses in Apple

**DOI:** 10.3390/foods7020011

**Published:** 2018-01-23

**Authors:** Houda Banani, Leone Olivieri, Karin Santoro, Angelo Garibaldi, Maria Lodovica Gullino, Davide Spadaro

**Affiliations:** 1 Department of Agricultural, Forestry and Food Sciences (DISAFA), University of Torino, Largo Braccini 2, 10095 Grugliasco (TO), Italy; houda.banani@unito.it (H.B.); leone.olivieri@gmail.com (L.O.); karin.santoro@unito.it (K.S.); marialodovica.gullino@unito.it (M.L.G.); 2 Centre of Competence for the Innovation in the Agro-environmental Sector (AGROINNOVA), University of Torino, Largo Braccini 2, 10095 Grugliasco (TO), Italy; angelo.garibaldi@unito.it

**Keywords:** apple, *Botrytis cinerea*, essential oils, induced resistance, priming, PR genes

## Abstract

The efficacy of thyme and savory essential oils were investigated against *Botrytis cinerea* on apple fruit. Apples treated with thyme and savory essential oils showed significantly lower gray mold severity and incidence. Thyme essential oil at 1% concentration showed the highest efficacy, with lower disease incidence and smaller lesion diameter. The expression of specific pathogenesis-related (PR) genes PR-8 and PR-5 was characterized in apple tissues in response to thyme oil application and *B. cinerea* inoculation. After 6 h of pathogen inoculation, thyme essential oil induced a 2.5-fold increase of PR-8 gene expression compared to inoculated fruits. After 24 h of inoculation, PR-8 was highly induced (7-fold) in both thyme oil-treated and untreated apples inoculated with *B. cinerea*. After 48 h of inoculation, PR-8 expression in thyme-treated and inoculated apples was 4- and 6-fold higher than in inoculated and water-treated apples. Neither thyme oil application nor *B. cinerea* inoculation markedly affected PR-5 expression. These results suggest that thyme oil induces resistance against *B. cinerea* through the priming of defense responses in apple fruit, and the PR-8 gene of apple may play a key role in the mechanism by which thyme essential oil effectively inhibits gray mold in apple fruit.

## 1. Introduction

Apple is one of the most important fruits in international trade; however, it can suffer from severe postharvest losses during long distance transport and storage. *Botrytis cinerea*, which causes gray mold, is one of the main pathogens causing postharvest losses of apples, especially for its conidial germination and mycelium growth at storage temperatures as low as 0 °C [1].

Gray mold control strategies mainly rely on chemical treatments; however, the use of synthetic fungicides is limited by the emergence of resistant strains. Following intensive use, resistance to thiabendazole has been reported in *Botrytis* populations [2]. In addition, public concern over human health and the environmental impact of pesticides have generated interest in developing effective and non-toxic approaches to control postharvest diseases of fruit [3,4].

Plant essential oils are gaining interest due to their apparently safe nature and their potential effectiveness as biopesticides for crop protection. On pome and stone fruit, as on table grapes, many plant essential oils have been effective against brown rot and gray mold rot [5,6]. Thyme and savory oils contain thymol, carvacrol, and p-cymene as volatile compounds, which have shown high antifungal activity [7,8,9]. The mechanism of activity of these promising fungicidal natural compounds has not been completely elucidated. However, their effectiveness was related to their components with phenolic structures, like carvacrol, eugenol, and thymol, which are highly active against pathogens, and their efficacy is often due to the synergy of different chemical components [9].

Other studies described their role in the induction of host resistance against pathogens. Induced resistance is a promising strategy to enhance a host’s defense capacity after treatment with biotic- or abiotic-inducing agents, which provides long-term systemic resistance to a broad spectrum of pathogens and pests [10,11]. Upon treatment with microorganisms or other resistance inducers, many hosts develop an enhanced capacity to activate defense responses, a phenomenon called priming [12]. Thymol could enhance antioxidant levels, enzymatic and non-enzymatic systems, which induce an increase of the fruit resistance to pathogens and a decrease of their physiological degradation [13]. Resistance inducers typically stimulate the expression of a family of genes in the host, defined as pathogenesis-related (PR) genes, which are involved in the defense response to pathogens [14]. In apple, PR-5 and PR-8 were identified and shown to be induced in response to different resistance inducers [15]. PR-5 is a thaumatin-like protein [16], while PR-8 codes for a class III chitinase [15]. However, no information is available regarding the involvement of PR genes induction in the biocontrol activity of plant essential oils against postharvest pathogens.

The objectives of this work were to evaluate the efficacy of important plant essential oils against *B. cinerea* in stored apple fruit, and then to investigate whether the efficacy of the oil application is associated with the priming of defense responses in apples.

## 2. Materials and Methods

### 2.1. Food Material and Microorganism

Apples (*Malus x domestica* Borkhausen ‘Red Fuji’) were harvested from a commercial orchard in Piedmont, Italy. Fruits were surface-sterilized and punctured with a sterile plastic tip. A strain of *Botrytis cinerea* Persoon was isolated from rotten apples and tested for its virulence by inoculation in artificially wounded apples. The strain was maintained on PDA (Potato Dextrose Agar, Merck, Darmstadt, Germany) slants at 4 °C and the spores were harvested after the pathogen was incubated on PDA in Petri dishes at 25 °C for 7 days. The strain was used throughout the experiments at a concentration of 10^5^ conidia/mL determined by a Bürker chamber (Knittel).

### 2.2. Essential Oils

Essential oils from savory (*Satureja montana* L.) and thyme (*Thymus vulgaris* L.) were purchased as commercial preparations with 99% purity from Soave (Turin, Italy). The compositional analyses were performed using a gas chromatograph Shimadzu (Kyoto, Japan) GC-2010 Plus equipped with a mass spectrometer GCMS-QP (Shimadzu, Kyoto, Japan) and a split-splitless injector (Shimadzu, Kyoto, Japan) [17]. A 10% stock emulsion (10% essential oil, 88% sterilized water, and 2% Tween 20, Merck, Darmstadt, Germany) was prepared from each essential oil. Emulsions of 1%, 0.5%, and 0.1% (*vol*/*vol*) were prepared for each essential oil application by diluting the stock emulsion with distilled water. All the resultant emulsions were shaken for 30 s before application to ensure a homogeneous essential oil mixture.

### 2.3. Disease Control Efficacy

The control activity of plant essential oils against *B. cinerea* strain was assessed as follows: 30 µL of each treatment was pipetted into apple wounds. Water served as a control. Twelve hours later, 30 μL of pathogen conidial suspension was inoculated into each wound. Also, a standard chemical (thiabendazole, Tecto 20S, Cerexagri Italia srl, San Carlo di Cesena (FC), Italy; 19.7% active ingredient, a. i.) was employed at 0.3 mg a. i. mL^−1^. Fruits were incubated at 23 °C. Rot incidence and diameter were measured 6 days after inoculation. Each treatment contained three replicates with 10 fruits per replicate and the experiment was performed three times. Wounded apples were treated with thyme (1%) in each wound. Twelve hours later, wounds were inoculated with *B. cinerea*. Apples treated with thyme or inoculated with *B. cinerea* were included to compare PR gene expression. Sterile distilled water served as a control. Apple tissue samples (exocarp and mesocarp) were obtained from 20 fruits stored at 23 °C at 6, 24, and 48 h post inoculation (hpi).

### 2.4. RNA Extraction and RT-qPCR

Total RNA was extracted from apple samples using an RNeasy^®^ Extraction Kit (Qiagen, Hilden, Germany), then treated with TURBO DNase (Thermo Fisher, Waltham, MA, USA). Absence of genomic DNA contamination was confirmed by PCR amplification of the elongation factor 1 α (EF1α) gene using a One Step RT-PCR Kit (Qiagen). First-strand cDNA was synthesized using an iScript cDNA Synthesis Kit (Bio-Rad, Hercules, CA, USA).

Reverse transcriptase (RT)-qPCR was performed in triplicate on cDNA obtained from each biological replicate using 2× Power SYBR Green Supermix (Bio-Rad). Amplification and detection were carried out in an iCycler (Bio-Rad), set up with initial denaturation at 95 °C for 10 min followed by 40 cycles comprising a denaturation step at 95 °C for 15 s and an annealing step at 52 °C for PR-8 and at 60 °C for PR-5 for 1 min. Primers PR8-F (5′-GCCACTGCAACCCCGCTAGT-3′), PR8-R (5′-GCGGGCGCGAATCTGACTGA-3′), PR5-F (5′-CAAGCAGCTTCCCTCCTCGGC-3’), and PR5-R (5′-GCCCCAGAAGCGACCAGACC-3’) were used to optimally amplify PR-8 and PR-5 gene sequences. Transcript levels of the elongation factor 1 (EF1α) served as an internal standard, and the primers used were EF1α-F (5′-GACATTGCCCTGTGGAAGTT-3′) and EF1α-R (5′-GGTCTGACCATCCTTGGAAA-3′) [18]. The expression ratio was calculated as 2−^ΔΔCT^, where ΔΔCT represents ΔCTsample—ΔCTcontrol [19], and values were normalized to those of control water at each time point.

### 2.5. Statistical Analysis

Statistical analysis was performed with SPSS version 20.0 (SPSS Inc., Chicago, IL, USA). Data obtained in all experiments were analyzed using analysis of variance (ANOVA). Means of treatment results were separated at the 5% significance level by using Duncan’s multiple range tests. Values are presented as the mean ± SD (standard deviation of the mean). Results are the mean of three independent experiments.

## 3. Results and Discussion

### 3.1. Efficacy of Essential Oils in the Control of Botrytis cinerea on Apples

**Figure 1:**
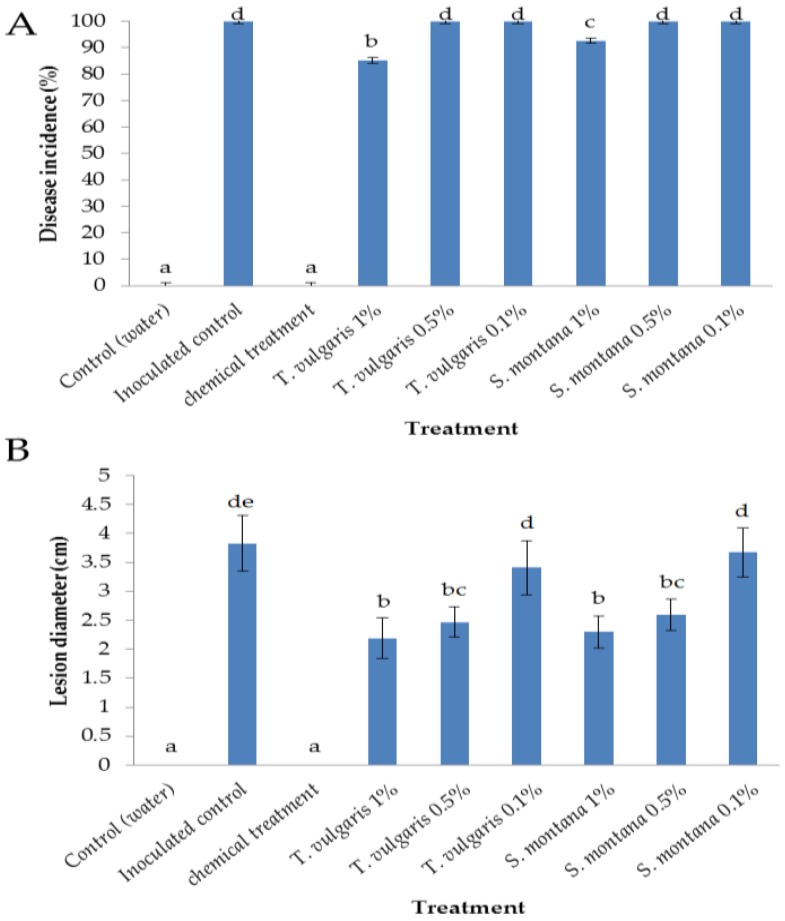
Antifungal activity of essential oils of *Satureja montana* and *Thymus vulgaris* applications at different concentrations (1%, 0.5%, and 0.1%) expressed as disease incidence (**A**) and lesion diameter (**B**) caused by *B. cinerea* on apples. Vertical lines represent the standard error for the average of three biological replicates. Applications followed by different letters are statistically different following Duncan’s multiple range test (*p* < 0.05).

Disease incidence and the diameter of lesions of *B. cinerea* rots were measured in ‘Red Fuji’ apples treated with two plant essential oils (Figure 1). Six days after pathogen inoculation, gray mold rot diameter and disease incidence on apples treated with essential oils of *T. vulgaris* and *S. montana* were significantly diminished as compared to the control. The highest disease reduction was obtained when essential oils were applied at the highest concentration (1%), especially thyme, which showed a disease incidence and rot diameter of about 85% and 21 mm, respectively, compared to 100% and 38 mm of the inoculated control.

Thyme essential oil, and its major components thymol and carvacrol, showed antifungal activity against plant pathogenic fungi [20]. Moreover, the inhibitory effect of thyme essential oil against *B. cinerea* on different apple cultivars was reported by Lopez-Reyes et al. [21], who confirmed that its efficacy is cultivar- and storage time-dependent. The phytotoxicity of the essential oils tested on apples was very low, as determined in previous experiments [21]. A certain level of toxicity to human and animal cell lines in vitro has been reported for some essential oils when used at high concentrations, but it was not reported at the concentrations tested in these experiments [22].

In the present study, the efficacy of thyme essential oil could also be related to the induction of host resistance, since the oil was applied 12 h before *B. cinerea* apple inoculation. However, additional molecular studies were accomplished to prove this hypothesis.

### 3.2. Effect of Thyme Oil Application and B. cinerea Inoculation on Defence Genes Expression in Apple Fruit

**Figure 2:**
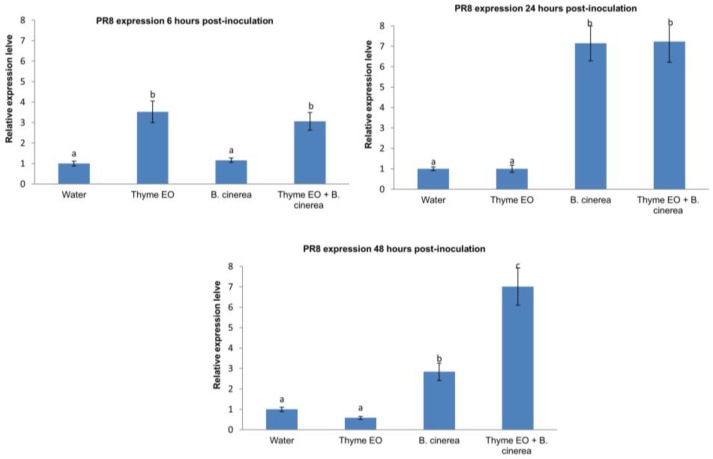
Expression of pathogenesis-related gene PR-8 in apple fruit in response to wounding, thyme essential oil, and *B. cinerea* at different time points (6 h, 24 h, and 48 h). Vertical lines represent the standard error for the average of three biological replicates. Different letters above the columns indicated a significant difference determined by Duncan’s multiple comparison test (*p* < 0.05).

**Figure 3:**
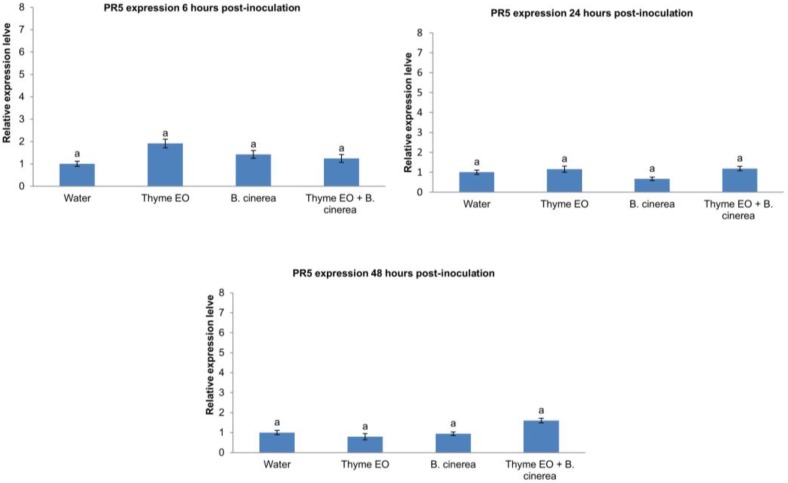
Expression of pathogenesis-related gene PR-5 in apple fruit in response to wounding, thyme essential oil, and *B. cinerea* at different time points (6 h, 24 h, and 48 h). Vertical lines represent the standard error for the average of three biological replicates. Different letters above the columns indicated a significant difference determined by Duncan’s multiple comparison test (*p* < 0.05).

In order to analyze the molecular mechanisms involved in thyme-induced resistance in apple, the expression of the two defense-related genes, PR-8 and PR-5, was analyzed in treated apples at different time points (Figure 2 and Figure 3).

After 6 h of pathogen inoculation, thyme essential oil induced a 2.5-fold increase of PR-8 gene expression compared to the negative control (wounded fruits treated with water), while no significant upregulation of PR-8 expression was observed in inoculated fruits.

After 24 h of inoculation, PR-8 expression in thyme-treated apples was similar to the negative control, whereas PR-8 was highly induced (7-fold) in both thyme-treated and untreated apples inoculated with *B. cinerea*. The early induction of PR-8 expression was no longer significant in thyme oil-treated fruit at 36 h after the essential oil treatment.

At this time point, PR-8 was induced similarly in inoculated untreated fruit and in inoculated treated fruit. This could be related to the early response of apple, which tries to counteract the penetration of *B. cinerea* mycelium. In a recently performed experiment on the germination of *B. cinerea* conidia on apple at 20 °C [23], the time points for germination were set at pregermination (1 h), postgermination (2.5 h), appressoria formation (4 h), and early mycelium (15 h). Therefore, we could presume that at 6 h, the pathogen has not yet penetrated the fruit, and thus PR-8 is not induced in inoculated fruit, while at 24 h the pathogen has already penetrated and the first mycelium is growing. The higher induction of PR-8 in inoculated fruit at 24 h compared to 6 h could be explained by the fungal development in the host fruit, since *B. cinerea* conidia formed appressorium-like terminal thickenings within 4 h of incubation, indicating the initiation of penetration [23]. The fruit induced PR-8 as an early response against the pathogen. Previous studies also showed that certain postharvest infections can induce host resistance [24].

Therefore, after penetration into the host tissue during the first 24 h after inoculation, *B. cinerea* could produce elicitors either directly or indirectly that are able to trigger a defense response strong enough to significantly increase the resistance of the fruit to a subsequent pathogen attack [23].

After 48 h of inoculation, PR-8 expression in apples treated with thyme oil and inoculated with the pathogen was 4- and 6-fold greater than that in inoculated and water-treated apples. This is the most significant effect, as 48 h after pathogen infection inoculated fruit no longer show an increase of PR-8 expression, while the response in fruits treated with oil and inoculated with *B. cinerea* is significantly higher.

Thyme essential oil influenced PR-5 expression during the trial, as shown in Figure 3. In particular, PR-5 expression was significantly increased after 6 h in apples treated with thyme essential oil with respect to the other treatments. After 24 h, PR-5 expression was similar in all fruits, while at 48 h the expression level was significantly higher in apples inoculated with *B. cinerea* and treated with thyme essential oil.

The treatment of plants and fruits with different inducers, such as cell walls, plant extracts, essential oils, compounds of biological origin, and synthetic chemical compounds, was shown to trigger resistance to pathogen attack locally and systemically [25]. There are two types of host induced resistance: induced systemic resistance (ISR) and systemic acquired resistance (SAR). SAR requires the signal molecule salicylic acid (SA) and is associated with the accumulation of pathogenesis-related (PR) proteins, which are thought to contribute to resistance [26]. Thyme essential oil demonstrated the ability to trigger defense response in avocado fruit. Thyme oil was applied through fumigation and induced the expression of β-1,3-glucanase and chitinase genes. The mRNA levels of the two enzymes were shown to be higher in fumigated fruit especially in the ripened stage. Fumigation with thyme oil caused the initiation of the induced defense response at the enzymatic and transcript level that made avocados less susceptible to anthracnose development, resulting in a treatment as effective as prochloraz treatment [27]. β-1,3-glucanases are PR-2 proteins able to damage the cell wall of many pathogenic fungi, while chitinase acts on C-bonds in chitin, an important and common component of the fungal cell wall [28]. Similar results were obtained in peaches, though the host response varied depending on the cultivar [29]. On avocado fruit, thyme essential oil also increased ammonia-lyase and peroxidase levels as well as antioxidant enzymes [30,31].

In our study, thyme essential oil significantly induced apple resistance against *B. cinerea* since it favored the accumulation of the pathogenesis-related protein PR-8, which codes for a class III chitinase.

After 6 h of pathogen inoculation, thyme caused a low direct induction of resistance (PR-8 expression level was 2.5-fold higher than that in water-treated fruits) since this signal activated the plant to a state of alertness. After pathogen infection, fruits strongly and efficiently responded, showing a long-lasting period of protection (PR-8 expression level was 6-fold greater than that in water-treated fruits after 24 and 48 h of pathogen inoculation). This effect is also known as the ‘priming effect’, which is beneficial since it is considered as a host solution to the trade-off dilemma between disease protection and costs involved in direct defense activation, and therefore the costs become affordable only when the host is exposed to pathogen attack [32]. Similarly, *Candida oleophila* was reported to elicit a long-lasting activation of PR-8 at 24 and 48 h, but a high level of PR-8 protein was present even at 8 h. PR-8 expression was higher than that in fruit inoculated with *B. cinerea*, and it increased during the trial. The rapid host response to *C. oleophila* could be due to the general activation of apple fruit defense when exposed to microbes [17]. The difference in PR-8 expression level and speed of defense activation when the fruit was exposed to thyme essential oil could be attributed to the nature of the elicitors, which could induce biotic or abiotic stress.

In contrast, a less clear response was achieved for PR-5 expression. As for PR-8 expression, the PR-5 level was also increased with respect to the untreated, inoculated or inoculated and treated fruit after 6 h. After 48 h, the only increase in PR-5 expression was obtained in inoculated fruit treated with thyme essential oil. Conversely, the presence of the pathogen did not induce any increase in PR-5 levels. Nevertheless, the magnitude of variation was very slight and it would be difficult to attribute any role of this gene to the induction of resistance under the studied conditions. Similar results were reported in literature on apples inoculated with *B. cinerea* or treated with *C. oleophila*. After 8 and 24 h, PR-5 gene expression was stable and similar to that observed in the untreated control. Only after 48 h were PR-5 levels higher in *B. cinerea*-inoculated apples in comparison to other treatments. However, since the expression level was even lower than that at time 0, in that case it was also difficult to attribute any biological significance to the observed pattern of expression [17].

## 4. Conclusions

Thyme essential oil at 1% could be used as an efficient postharvest treatment against *B. cinerea* in stored apple. Based on gene expression results, it is postulated that one mechanism by which thyme effectively controls gray mold disease in apple fruit is through the induction of the PR-8 gene in apple fruit, besides its known antifungal activity. Deeper studies are required to understand the role of the PR-8 protein as a pathogen defense mechanism, its expression in various apple cultivars against a wide range of pathogens, and how specific pathogens may interfere with PR-8 expression.
